# Impetigo Leishmaniasis Previously Diagnosed as Crusty Impetigo: A Case Study

**DOI:** 10.7759/cureus.22492

**Published:** 2022-02-22

**Authors:** José Suarez, Margarita Rios, Dora Estripeaut, Adelys Reina

**Affiliations:** 1 Tropical Medicine, Gorgas Memorial Institute of Health Studies, Panama, PAN; 2 Sistema Nacional de Investigadores (SNI) Research, Secretaría Nacional de Ciencia, Tecnología e Innovación (SENACYT), Panama, PAN; 3 Infectious Disease, Hospital del Niño Dr. José Renan Esquivel, Panama, PAN; 4 Parasitology, Gorgas Memorial Institute of Health Studies, Panama, PAN

**Keywords:** atypical presentation, panama, leishmania panamensis, cutaneous leishmaniasis, impetigo

## Abstract

Cutaneous leishmaniasis is a zoonotic disease caused by several species of protozoa of the genus *Leishmania*. Cutaneous leishmaniasis classically presents as an ulcer with heaped edges, but it can also appear as nodular, scabbed, or plaque-like lesions. Its diagnosis requires confirmatory laboratory tests such as a smear, culture, and polymerase chain reaction. However, atypical presentations represent a diagnostic challenge in Tropical Medicine. For instance, localized cutaneous leishmaniasis (LCL) resembles bacterial and fungal tropical dermatological infections. Atypical presentations require an experienced clinician, epidemiological knowledge, and proper diagnostic tests. We present a case of a 10-year-old male who showed classic impetigo-like symptoms, which did not improve with topical or systemic antibiotic therapy. After a thorough case review, the patient was diagnosed with LCL. Therefore, epidemiological and clinical evaluation is crucial when diagnosing, especially in patients who live or have travelled to leishmaniasis-endemic areas.

## Introduction

Leishmaniasis is an infection of the mononuclear phagocytic system caused by flagellated protozoa from Leishmania genera. These parasites are transmitted by Diptera insects primarily from the genus Phlebotomus in the Old World and Lutzomyia in the New World. Depending on the tropism of the parasite, clinical presentation patterns include viscera-tropic, dermatotropic, and mucotropic disease states. In addition, cutaneous disease forms depend on the host’s immune response, ranging from a mild, isolated cutaneous disease to more severe forms, such as a diffuse, mucocutaneous, and disseminated disease [1,2]. This range of the disease partly results from the individual cellular immune response of the host’s lymphocytic types, namely, T-helper lymphocytes 1 and 2 (Th1 and Th2, respectively) [1].

The World Health Organization classifies leishmaniasis as Category I, which means an emerging or poorly controlled disease. Approximately 350 million people are at risk for leishmaniasis, 12 million people are infected, and one million new cases are reported annually [ 1,3,4]. In particular, localized cutaneous leishmaniasis (LCL) can imitate bacterial and fungal tropical dermatological infections. Atypical presentations of leishmaniasis require an experienced clinician, epidemiologic knowledge, and diagnostic tests such as smear, culture, and polymerase chain reaction (PCR) [1-5], indicating a diagnostic challenge in Tropical Medicine. Here, we present a clinical case of a 10-year-old male with seemingly classic impetigo who was subsequently diagnosed with LCL.

This article was previously presented as a poster at the 2018 National Infectious Diseases meeting in Panama City, Panama, in October 2018.

## Case presentation

**Figure 1:**
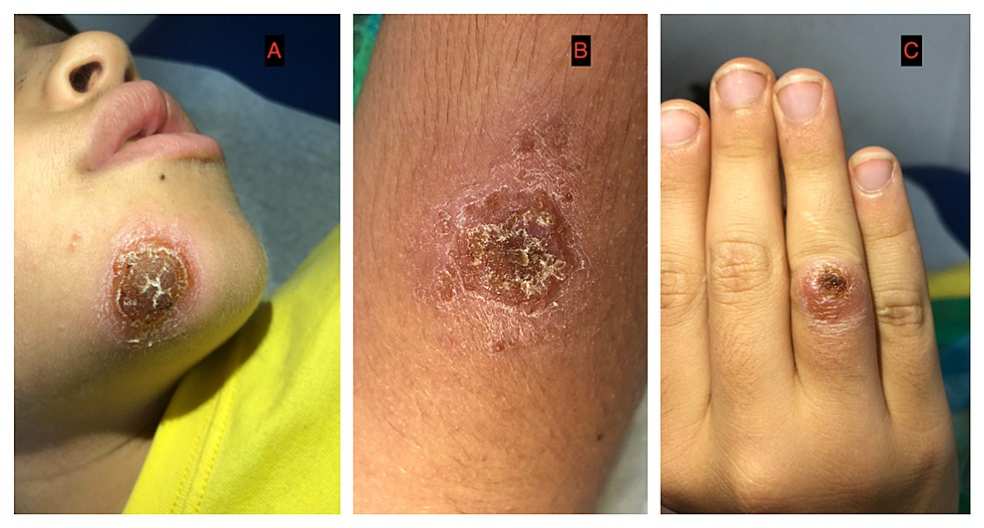
A-C. Initial presentation of cutaneous lesions diagnosed as impetigo with no improvement after systemic and topical antibacterial therapy.

A 10-year-old male patient from the Bocas del Toro Islands of Panama presented with fever for two weeks and multiple, irregular crops of irritable superficial plaques with hyperpigmentation and some with the honey-like surface on the chin, arm, forearm, right hand, and the inner side of the left thigh that have progressively worsened over two months. He was treated with one million units of intramuscular procaine penicillin daily for five days, followed by 40 mg/Kg/day of amoxicillin (250 mg/5 ml) every eight hours for seven days. However, the clinical manifestations did not improve. Upon consultation with our clinic, the diagnosis of impetigo seemed correct; thus, he was treated for methicillin-susceptible Staphylococcus aureus impetigo with 30mg/kg/day of cefadroxil (250 mg/5 ml) orally every 12 hours for 10 days. Nasal swabs were collected for the culture of *Staphylococcus spp.*, which was then found to be negative. After 10 days, the patient returned with no improvement in his clinical condition. His lesions were thoroughly cleaned and unroofed, revealing chronic ulcers with a wet base and high edges. Hence, topical anesthesia was administered, and a sample was collected for smear examination with Giemsa staining, culture, and polymerase-chain-reaction (PCR) for *Leishmania spp*. Figure 1 shows the initial presentation of cutaneous lesions. 

**Figure 2:**
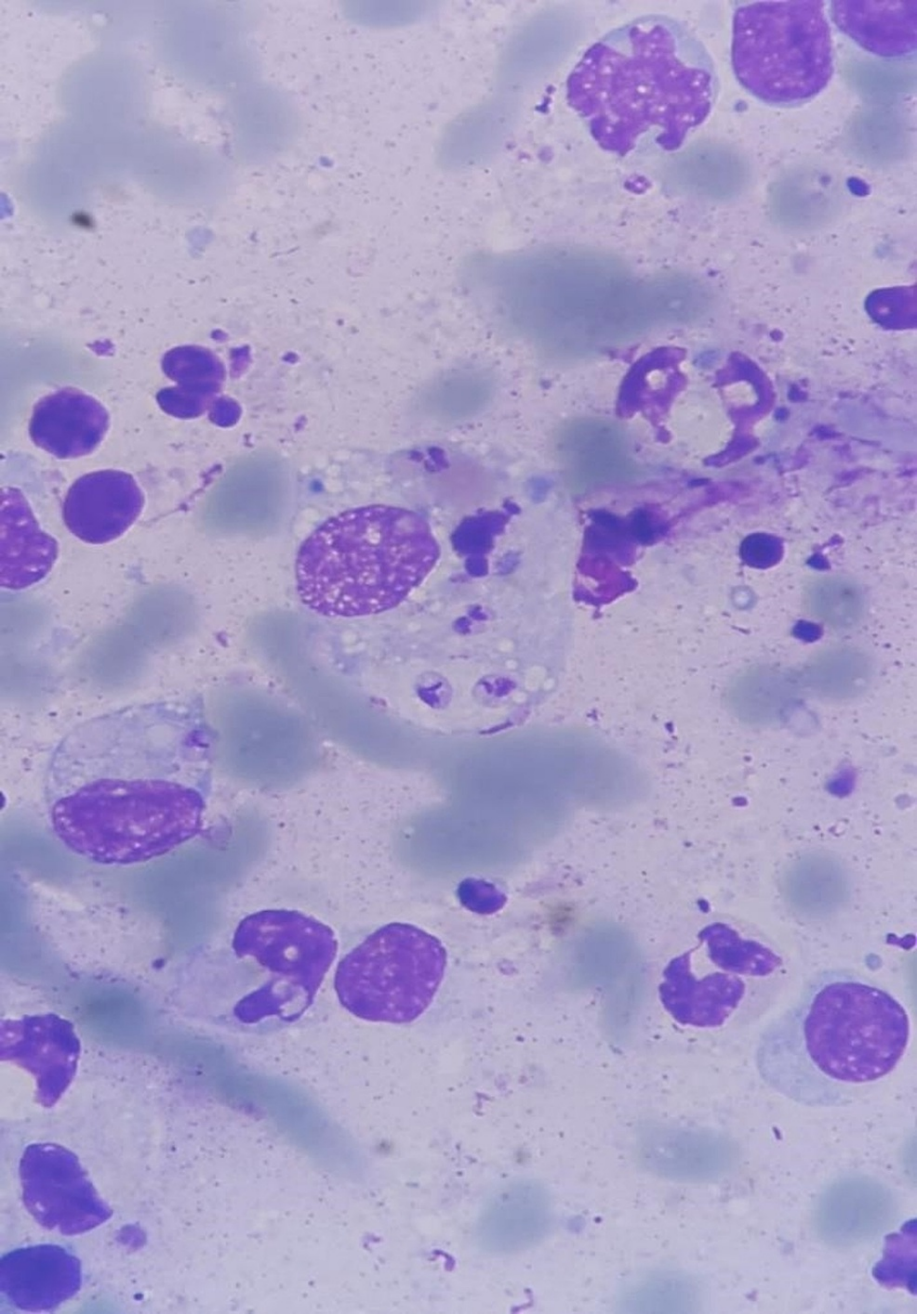
Apposition smear, Giemsa stain, 100×.

**Figure 3:**
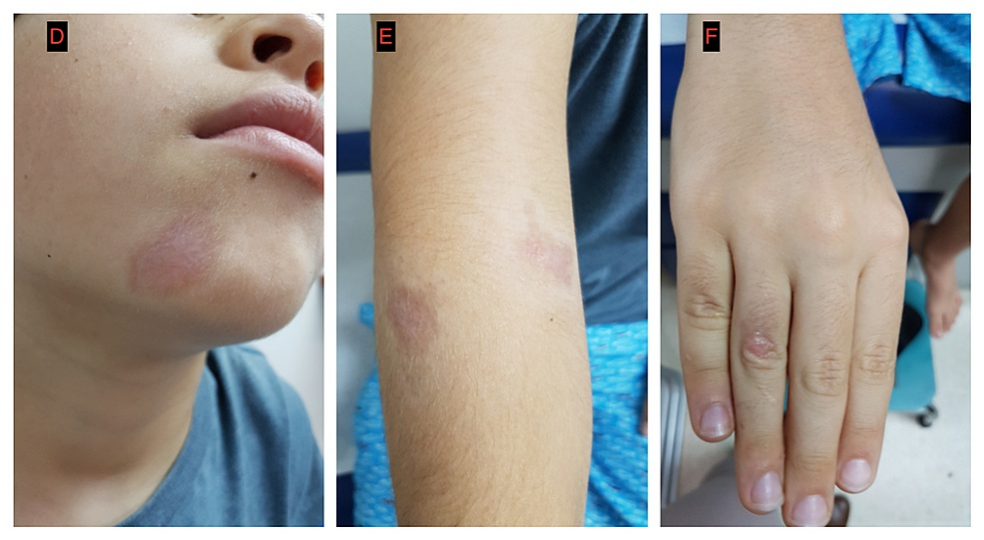
D-F. Improvement of cutaneous lesions after treatment with pentavalent antimonial (Glucantime ©).

By smear examination, amastigotes were detected (Figure 2). Additionally, PCR and culture-confirmed *Leishmania Viannia panamensis* as the etiological agent. Treatment was initiated with intramuscular meglumine antimoniate at 20 mg/kg/day for 20 days. After 11 days, the lesions were completely healed (Figure 3).

## Discussion

LCL should be considered as a differential diagnosis of nonhealing ulcers and other types of nonhealing lesions among individuals who reside or who have traveled on endemic areas. Unfamiliarity with the wide spectrum of cutaneous manifestations among healthcare providers represents an enormous challenge, as demonstrated in recent reports [3]. In the study of Paniz-Mondolfi et al. [3], 48% of patients with LCL were only diagnosed after a non-medical relative suggested the possibility of this parasitic infection or after the patient sought specific care for leishmaniasis.

The diagnosis of LCL should take into consideration the wide spectrum of its clinical cutaneous manifestations that mimic many different infectious diseases as well as malignant skin pathologies in some cases (Table 1). Clinical suspicion of nonhealing cutaneous lesions requires an epidemiological understanding of geographic regions at risk of transmission and a confirmatory diagnosis through specific histopathological assessment and molecular testing for the presence of amastigotes and/or nucleic acids by PCR, respectively [4]. In Central and South America, with an incidence of 18.37 per 100,000 inhabitants for LCL [5], the endemic infectious diseases included in differential diagnoses are impetigo, mycobacterial infections (leprosy, Buruli ulcer, and Mycobacterium ulcerans), furunculosis, sporotrichosis, chromomycosis, paracoccidiomycosis, botryomycosis, nocardiosis, and actinomycetoma. Noninfectious diseases that mimic LCL include basal cell carcinoma, squamous cell carcinoma, lymphocytoma cutis, arthropod bites, and pyoderma gangrenosum [4,6]. Other entities such as Yaws caused by *Treponema pallidum* *pertenue* should be considered in Africa and Southeast Asia and blastomycosis in North America.

**Table 1:** Classic and atypical forms of Localized Cutaneous Leishmaniasis. Taken from [7-8].  Modified by Ríos and Suarez.

Clinical presentation	General characteristics
Acute paronychial	Painful swelling, erythema, and crusting of nail folds.
Chancriform	Painless punched-out ulcers with reddish-blue indurated margins and a granulating floor without regional lymphadenopathy.
Palmoplantar	Painless, non-pruritic, roughly circular, solitary, crusted, and scaly plaques.
Zosteriform	The roughly linear arrangement of satellite papules around the main lesions on the trunk.
Erysipeloid	Erythematous, indurated, ill-defined lesion.
Chromomycoid	Warty skin lesions with a horny appearance and firm consistency, reminiscent of warty tuberculosis.
Sporotricoid	A skin lesion that is accompanied by lymphangitis and a series of nodules that soften and then begin to ulcerate, appearing staggered in the lymphatic trunk.
Gomoid or syphiloid	A dermal nodule that appears softens, opens, and sloughs, constituting ulceration with a crateriform and fetid bottom and edges cut to a peak and of firm consistency.
Pyodermoid	With furunculosis and impetigo forms, similar to boils and impetigo.
Epitheliomatoid	Vegetating lesion displaying a torpid evolution with a granular and proliferating background and without infiltrated edges of firm consistency and adhering to the deep planes.
Leishmanic frambuesoma	A red vegetative, strawberry-like lesion that bleeds with the slightest touch and subsequently ulcerates. It is clinically indistinguishable from the bulbous lesion.

Our case illustrates that LCL can take many forms; the classical classification is still relevant in places where the incidence of LCL is high and modern diagnostic tools are often needed to obtain the appropriate diagnosis. PCR was used for the molecular diagnosis, for the amplification, a total reaction of 50 ul was used using 3 uL (0.6uM) of the primers B and B2, which were employed to amplify the DNA of an entire minicircle of the kinetoplast in Leishmania subgenus Viannia [9,10] using 25 ul of PCR mix, 2 ul of MgCl2 (1mM), 12 ul of molecular quality water and 5 ul of the sample.

The thermocycling cycles were: initial denaturation 95°C for six minutes followed by five cycles at 95°C for 30 seconds, 64.5°C for two minutes and 72°C for one minute followed by 35 cycles at 95°C for 30 seconds, 64°C for one minute and 72°C for one minute and a final extension at 72°C for 10 minutes, 4° for an indefinite amount of time. The 750bp product was then visualized on a 1.5% agarose gel.

The Leishmania species was characterized using polymerase chain reaction-restriction fragment length polymorphism (PCR-RFLP). The product amplified by PCR-RFLP is a band of 1286 bp, which is digested with a restriction enzyme called HaeIII. If the pattern resembles that of the species of the subgenus *Viannia*, this band is subsequently digested by either Bcc I or Rsa I, which produces a particular pattern for the different species of this subgenus *Viannia* [11]. 

LCL should be considered in atypical skin lesions in endemic countries such as Panama. While the classic appearance of a flat, wet base with heaped edges is often for LCL, more atypical presentations frequently occur [12].

## Conclusions

LCL is a widely spread parasitic infection with complex epidemiological behavior, especially among children. Thus, LCL diagnosis requires not only the knowledge of the diversity and complex pathophysiology of infectious entities in the tropics but also awareness of the multiple presentations of LCL. In countries where leishmaniasis is endemic, the classic descriptions of this entity are still valid and can be a tool for diagnosis and management.
